# Reliability of bioreactance and pulse power analysis in measuring cardiac index during cytoreductive abdominal surgery with hyperthermic intraperitoneal chemotherapy (HIPEC)

**DOI:** 10.1186/s12871-023-01988-3

**Published:** 2023-01-31

**Authors:** Laura Anneli Ylikauma, Mari Johanna Tuovila, Pasi Petteri Ohtonen, Tiina Maria Erkinaro, Merja Annika Vakkala, Heikki Timo Takala, Janne Henrik Liisanantti, Timo Ilari Kaakinen

**Affiliations:** 1 Research Group of Surgery, Intensive Care Unit, Anaesthesiology and Intensive Care Medicine, Medical Research Center Oulu, Oulu University Hospital and University of Oulu, PL 21, 90029 OYS Oulu, Finland; 2 Research Service Unit, Oulu University Hospital, Oulu, Finland; 3 Department of Surgery, Oulu University Hospital, Oulu, Finland

**Keywords:** Bioreactance, Cardiac output, Hyperthermic intraperitoneal chemotherapy, Hemodynamic monitoring, Pulse power analysis

## Abstract

**Purpose:**

Various malignancies with peritoneal carcinomatosis are treated with cytoreductive surgery and hyperthermic intraperitoneal chemotherapy (HIPEC). The hemodynamic instability resulting from fluid balance alterations during the procedure necessitates reliable hemodynamic monitoring. The aim of the study was to compare the accuracy, precision and trending ability of two less invasive hemodynamic monitors, bioreactance-based Starling SV and pulse power device LiDCOrapid with bolus thermodilution technique with pulmonary artery catheter in the setting of cytoreductive surgery with HIPEC.

**Methods:**

Thirty-one patients scheduled for cytoreductive surgery were recruited. Twenty-three of them proceeded to HIPEC and were included to the study. Altogether 439 and 430 intraoperative bolus thermodilution injections were compared to simultaneous cardiac index readings obtained with Starling SV and LiDCOrapid, respectively. Bland-Altman method, four-quadrant plots and error grids were used to assess the agreement of the devices.

**Results:**

Comparing Starling SV with bolus thermodilution, the bias was acceptable (0.13 l min^− 1^ m^− 2^, 95% CI 0.05 to 0.20), but the limits of agreement were wide (− 1.55 to 1.71 l min^− 1^ m^− 2^) and the percentage error was high (60.0%). Comparing LiDCOrapid with bolus thermodilution, the bias was acceptable (− 0.26 l min^− 1^ m^− 2^, 95% CI − 0.34 to − 0.18), but the limits of agreement were wide (− 1.99 to 1.39 l min^− 1^ m^− 2^) and the percentage error was high (57.1%). Trending ability was inadequate with both devices.

**Conclusion:**

Starling SV and LiDCOrapid were not interchangeable with bolus thermodilution technique limiting their usefulness in the setting of cytoreductive surgery with HIPEC.

## Introduction

Cytoreductive surgery (CRS) with hyperthermic intraperitoneal chemotherapy (HIPEC) has become a standard treatment for various malignancies with peritoneal carcinomatosis [1]. It is indicated in pseudomyxoma peritonei, peritoneal mesotheliomas and colorectal cancer with peritoneal carcinomatosis [2]. It has allowed to cure many patients that previously were treated with palliation, and it is the only curative treatment for peritoneal surface malignancies [2].

CRS consists of major surgical resections [2, 3]. It is followed by HIPEC, during which warmed (42 °C) chemotherapies are infused inside the abdominal cavity [3]. CRS with HIPEC is considered high-risk surgery due to major fluid losses, long duration of the surgery and thermal stress [2–4]. Aggressive fluid resuscitation is often needed, but fluid overdose should be avoided since it worsens the outcome [2]. Hemodynamic monitoring with mini-invasive monitors is recommended in the literature to help assess the volume status during CRS with HIPEC [1].

In clinical settings, measuring cardiac output (*CO*) by using bolus thermodilution technique with a pulmonary artery catheter (TDCO) is considered the gold standard [5–7]. Since pulmonary artery catheter (PAC) is invasive and can potentially cause harm to the patients, less invasive *CO* monitors have been developed [5, 8]. There are various different devices commercially available, but their reliability is still questionable [9]. Starling™ SV (CMM-ST5, 2017-12-01, version 5.2, Cheetah Medical, Newton, Massachusetts, USA) is a completely noninvasive, continuous *CO* monitor, that utilizes transthoracic bioreactance technique [9]. LiDCOrapid (LiDCOrapid V2.03–318, LiDCO, London, UK) is a mini-invasive, continuous *CO* monitor and it is based on arterial pressure waveform analysis [10]. We used cardiac index *(CI)* instead of *CO* in our study. *CI* is calculated by dividing *CO* by patient’s body surface area.

In this study we compared the accuracy, precision and trending ability of two less invasive *CO* monitors, noninvasive Starling SV and mini-invasive LiDCOrapid, with TDCO in patients undergoing CRS with HIPEC.

## Material and methods

Ethical approval of this study (27/2018) was provided by the Ethical Committee of Oulu University Hospital, Oulu, Finland (Chairperson Prof J. Mäkelä) on 20 April 2018, and the amendment was approved on 14 October 2019. The prospective single-center observational method comparison study was conducted according to the principles of the Helsinki declaration. Written informed consent for participation in the study was obtained from all the patients. Our exclusion criteria were the refusal of the patient to attend the study and the withdrawal from HIPEC after clinical evaluation during surgery.

Prior to the induction of general anaesthesia, a thoracic epidural catheter was inserted and tested with a combined bolus of lidocaine and adrenalin. The anaesthesia induction was performed with propofol, remifentanil and rocuronium, and maintained with desflurane, remifentanil and rocuronium. During HIPEC, desflurane was replaced with propofol infusion because of its neuroprotective features [11]. Epidural analgesia was maintained with boluses of fentanyl and 0.9% saline or with an infusion consisting of fentanyl, levobupivacaine and 0.9% saline. Vasoactive agents used were norepinephrine and dobutamine, if needed.

Esophageal, urinary bladder and peripheral skin temperatures were measured continuously throughout the operation, in addition to the direct blood temperature monitoring with PAC. Normothermia was preserved by using warm fluids, a thermal blanket and a noninvasive temperature management system (Arctic Sun®, Bard Medical, Covington, GA). During the HIPEC phase temperatures from the upper and lower stomach were also monitored. The fluid balance was carefully evaluated during the operation and hypovolemia was treated with balanced crystalloid solutions, albumin, fresh frozen plasma and packed red blood cells, as needed. Prior to HIPEC the patients received 10 mg of i.v. furosemide to increase urine output.

After the cytoreductive stage was performed, the intraperitoneal cavity was filled with 43–44 °C 0.9% saline by The Belmont Hyperthermia Pump™ (Belmont Instrument Corporation, Billerica, MA, USA). When the targeted intra-abdominal temperature of 41 °C was reached, either Mitomycin C, divided into three proportions, or the combination of doxorubicin and cisplatin (for mesothelioma) was administered into the circulating intraperitoneal fluid. We used the open coliseum technique [4]. The duration of HIPEC was 90 minutes, and during that time the patients were actively cooled with the Arctic Sun, cold fluid infusions and cooling packs around the upper body and head. After HIPEC, the surgery was completed and the patients were transferred to the intensive care unit (ICU) for postoperative care.

During the procedure, *CI* was monitored with TDCO and the study devices. We used *CI* instead of *CO* according to the clinical practice in our hospital, since it takes patient size into consideration. *CI* was calculated automatically by the monitors dividing *CO* by patient’s body surface area. Starling SV is a continuous, noncalibrated and noninvasive *CO* monitor. It is based on transthoracic bioreactance technique [9]. Four dual-electrode stickers are placed on the chest wall, two on the left and two on the right side [12]. An alternating electrical current is produced through the thorax. Aortic blood flow creates a phase shift between the applied current and the measured thoracic voltage. This phase shift is related to *CO* [10, 12]. LiDCOrapid is a continuous, noncalibrated and mini-invasive *CO* monitor, and its method is called pulse power analysis. It utilizes an autocorrelation algorithm, PulseCO™, to calculate the stroke volume using the entire arterial pressure waveform [10]. The device does not need external calibration, since the vascular compliance of the patient is assessed by a nomogram and the estimated stroke volume is based on patient variables such as age, height and weight [10].

When the patients entered the operating room, we placed four dual-electrode stickers of Starling SV on the back of the patients [12]. An arterial line was inserted into the radial or brachial artery (BD™ Arterial Cannula 20 gauge, Becton Dickinson and Company, Franklin Lakes, New Jersey, USA). LiDCOrapid was connected to the patient monitor (Carescape B850 Monitor, GE Healthcare, Chicago, Illinois, USA). An 8.5 French introducer sheath was inserted in the right internal jugular vein, and a 7.5 French PAC (Criticath® SP5507U TD Catheter, Merit Medical, South Jordan, Utah, USA) was advanced into the pulmonary artery. The correct placement was confirmed by identifying and measuring the pulmonary capillary wedge pressure.

TDCO was measured approximately once in an hour during the operation depending on the stability of the patient hemodynamics. During hemodynamical disturbances measurements were performed more often, if necessary. With some patients, during stable conditions, the measurement interval was longer than 1 h. Each TDCO measurement was a mean of at least three 10 ml 0.9% saline bolus injections at room temperature [13]. Unreliable thermodilution curves were deleted based on their morphology and coherence compared to the other measurements. The TDCO measurements were not synchronized with the respiratory cycle [14]. At the start of Starling SV monitoring, the device calibrates itself automatically. Thereafter, manual recalibrations can be done every time the position of the patient changes, or if the signal becomes unreliable. The data from Starling SV was stored into its own database. The *CI* of Starling SV was recorded once a minute. As the time settings were synchronized with our electronic patient monitoring system, we could analyze the measurements performed at the same time as TDCO. One *CI* value with Starling SV is a mean of *CIs* measured during 1 min. To decrease potential error, the value was not accepted if it was significantly divergent from the values before and after. LiDCOrapid was not calibrated. Arterial line was zeroed in the beginning and every time the signal was unreliable confirming reliable data for LiDCOrapid. The data from LiDCOrapid was written down manually each time *CI* was measured with TDCO, and thereby we could make sure that the measurements were done simultaneously. The values were evaluated bedside, and if the LiDCOrapid values changed significantly during the evaluation, the value was not accepted until it was stabilized.

We divided our data into four phases. The first phase was before the initiation of CRS in the OR, the second phase during CRS, the third phase during HIPEC and the fourth phase after HIPEC in the OR. The study protocol ended when the patient was transferred to the ICU for postoperative care.

The sample size was calculated as recommended in the literature. We considered the data structure with multiple independent measurements within the subject [15]. The sample size calculations were based on the first 10 patients, from whom we took 121 measurements altogether. We calculated the mean *CI* and the standard deviation of TDCO and assumed that there was a 10% difference compared with the other two monitors. Noninferiority margin 0.30, alpha 0.05 and beta 0.10 were used, resulting in 428 measurement points.

All analyses were performed using SPSS® for Windows (IBM® Corp. IBM SPSS Statistics for Windows, Version 25.0. Armonk, NY: IBM Corp.) and SAS® for Windows (version 9.4 SAS Institute Inc., Cary, NC, USA). We present our summary statistics as medians with 25th to 75th percentiles unless stated otherwise, and two-tailed *P*-values are given.

The Bland-Altman plot was used to evaluate the mean bias for accuracy with limits of agreement (LOA) for precision between each test monitor and TDCO [7, 16–20]. Proportional bias was evaluated by calculating regression coefficients [7]. We declared the 95% Confidence Intervals (95% CI) for bias, LOA and regression coefficients [21]. For calculation of LOA, we used the method of multiple independent measurements within the subject [16, 17]. Precision was described also by reporting percentage errors (PE) with 95% CI [7, 22]. Percentage error was calculated according to Critchley and Critchley as two times standard deviation divided by mean *CI* [(2 x SD) / mean *CI*] [22]. Predefined targets for acceptable bias, LOA and PE were set according to the literature, resulting in 0.25 l min^− 1^ m^− 2^, 0.5 l min^− 1^ m^− 2^ and 30%, respectively [7, 22]. However, when interpretating the results it is important to take into consideration, that the clinical relevance of absolute target values depends on the magnitude of *CI*.

To evaluate the trending ability, we used the four-quadrant (4Q) plot, which consists of the changes of two consecutive *CI* measurements (delta *CI*) obtained with the reference method and the study monitor [7, 23]. The consecutive measurements were performed approximately every hour. We included the exclusion zone of 0.25 l min^− 1^ m^− 2^ as recommended in the literature. Points at the lower left and upper right quadrants reflect, that *CI* has either decreased or increased with both devices, respectively. However, the 4Q plot does not offer clinically usable cutoff values for defining the level of agreement nor does it estimate the magnitude of changes [7, 23].

Clinical concordance can be evaluated with error grid, where the boundaries are made from a clinical perspective helping clinical decision-making. The changes in *CI* with reference method are divided into three categories. The change is non-significant, if it is 5% or less. Moderate change is a change between 5 and 15%. The change is large if it is more than 15%. If the *CI* of experimental device change the same direction and to the same extent as reference method, the trending is good. All data pairs are used in the analysis without exclusion zone. Based on the 4Q plots, error grids with four zones were created. In zone 1 the change in *CI* measured with the study device and TDCO has either been positive or negative, and the extent of the change has been equal. These results lead to similar treatment interventions. In zone 2 both devices have detected similarly the direction of the change, but the extent of the change is not comparable. In zone 3 only one device has detected the change in *CI*, and in zone 4 the changes have been opposite, which may result in divergent treatment decisions [7].

## Results

We recruited 31 patients between May 2018 and September 2020. These patients had been evaluated preoperatively as having a high probability for CRS with HIPEC. In eight patients the HIPEC procedure was withheld perioperatively either because there was no need for it or because the disease was too widely spread. Eventually, we included 23 patients to our study. The patient characteristics are presented in Table 1. Most of the patients had either carcinomatosis from colorectal adenocarcinoma or pseudomyxoma peritonei. Median peritoneal cancer index (PCI) was 11. Altogether 439 and 430 simultaneous measurements were performed with Starling SV and LiDCOrapid, respectively, and they were compared to TDCO. The median number of measurements per patient was 17. We received 416 and 402 delta *CI* measurements with Starling SV and LiDCOrapid, respectively, which we compared with the delta *CI* measurements with TDCO and used for the 4Q plot.

**Table 1:** Patient characteristics (*n* = 23)

Age, years	57 (50 to 68)
Sex, male	14 (61)
BSA, m^2^	2.00 (1.73 to 2.25)
BMI, kg m^− 2^	26.3 (24.0 to 30.9)
Prior co-morbidities	
ASA physical status classification	
ASA 2	20 (87)
ASA 3	3 (13)
Hypertension	9 (39)
Type 2 diabetes mellitus	3 (13)
Asthma / COPD	4 (17)
Atrial fibrillation	1 (4)
Coronary artery disease	1 (4)
Previous valve procedure	2 (9)
Prior HIPEC-surgery	3 (13)
Medication prior to surgery	
Beta blocker	3 (13)
ACE inhibitor or AT II receptor inhibitor	7 (30)
Medical state prior to surgery	
Haemoglobin, g l^−1^	137 (121 to 148)
Thrombocytes, × 10^9^ l^− 1^	300 (236 to 356)
INR	1.0 (1.0 to 1.1)
GFR over 59 ml min^− 1^ 1.73 m^− 2^	23 (100)
Diagnose	
Colorectal adenocarcinoma	11 (48)
Pseudomyxoma peritonei	10 (44)
Mesothelioma	1 (4)
Goblet cell carcinoma of the appendix	1 (4)
Surgery	
Peritoneal cancer index	11 (4 to 26)
Chemotherapy used: mitomycin	22 (96)
Chemotherapy used: cisplatin and doxorubicin	1 (4)
Norepinephrine max dose, μg kg^−1^ min^− 1^	0.11 (0.08 to 0.15)
Dobutamine max dose, μg kg^−1^ min^− 1^	0.96 (0.00 to 1.69)
OR stay, min	770 (620 to 860)
ICU length of stay, days	1 (1 to 2)
Time at tertiary care hospital, days	15 (11 to 20)
Hospital mortality	0 (0)

The values given are medians with 25th and 75th percentiles, or number of patients, *n*, with percentages (%)

*BSA* Body surface area, *BMI* Body mass index, *COPD* Chronic obstructive pulmonary disease, *HIPEC* Hyperthermic intraperitoneal chemotherapy, *ACE* Angiotensin converting enzyme, *AT* Angiotensin, *INR* International normalized ratio, *GFR* Glomerular filtration rate, *ICU* Intensive care unit

**Fig. 1:**
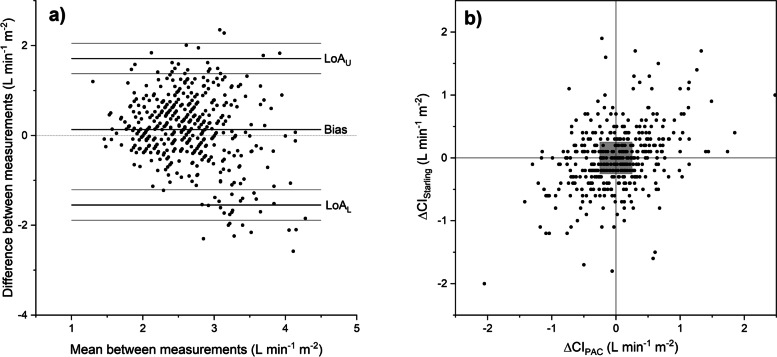
**a** The Bland-Altman analysis for cardiac index measurements with bioreactance-based Starling SV and the bolus thermodilution technique with a pulmonary catheter, all measurement points. The bias and LOA with 95% CIs are shown with lines. Exact numbers are seen in Table 2. **b** The trending ability of Starling SV evaluated with four-quadrant plot at all measurement points. The changes in consecutive cardiac index measured with Starling SV (ΔCI_Starling_) and the bolus thermodilution technique (ΔCI_PAC_) are plotted against each other

With each of the devices, TDCO, Starling SV and LiDCOrapid, the mean *CI* (with standard deviation) were 2.72 l min^− 1^ m^− 2^ (0.34), 2.64 l min^− 1^ m^− 2^ (0.43) and 3.02 l min^− 1^ m^− 2^ (0.64), respectively. Starling SV compared with TDCO was associated with a bias of 0.13 l min^− 1^ m^− 2^ (95% CI 0.05 to 0.20) at all measurement points. Standard deviation of the differences was 0.79. LOA were − 1.55 to 1.71 l min^− 1^ m^− 2^ and PE was 60.0% when all measurement points were regarded (Fig. 1a). The regression coefficient was − 0.25 l min^− 1^ m^− 2^. However, at phase 4 the bias was 0.52 l min^− 1^ m^− 2^ with a regression coefficient of − 0.45 l min^− 1^ m^− 2^, indicating a change in bias when *CI* changes. In the 4Q plot the changes in *CI* measured by Starling SV and TDCO were plotted against each other (Fig. 1b). The level of agreement in trending was assessed with error grids resulting in 32.5% of the measurement points being in zone 1. All the results between Starling SV and TDCO are shown in Tables 2 and 4.

**Table 2:** Results from cardiac index measurements made by Starling SV compared to bolus thermodilution technique with a pulmonary artery catheter

Starling SV	Bias (l min^− 1^ m^− 2^)	Bias 95% CI	LOA lower (l min^− 1^ m^− 2^)	LOA lower 95% CI	LOA upper (l min^− 1^ m^− 2^)	LOA upper 95% CI	Percentage error (%)	Percentage error 95% CI	Regression coefficient (l min^− 1^ m^− 2^)	Regression coefficient 95% CI
All *n* = 439	0.13	0.05 to 0.20	−1.55	− 1.89 to − 1.21	1.71	1.37 to 2.05	60.0	47.0 to 72.8	− 0.25	− 0.37 to − 0.13
Phase 1 *n* = 32	0.04	− 0.18 to 0.27	− 1.31	−1.79 to − 0.84	1.25	0.77 to 1.72	58.3	33.7 to 77.7	0.17	− 0.49 to 0.83
Phase 2 *n* = 273	0.11	0.02 to 0.19	−1.42	− 1.79 to − 1.05	1.63	1.26 to 2.01	56.9	44.2 to 73.0	− 0.16	− 0.30 to − 0.02
Phase 3 *n* = 64	− 0.16	− 0.39 to 0.07	− 2.11	− 2.71 to − 1.50	1.88	1.27 to 2.48	60.5	45.8 to 84.6	− 0.25	− 0.86 to 0.35
Phase 4 *n* = 70	0.52	0.37 to 0.68	−1.06	−1.49 to −0.63	1.88	1.45 to 2.31	50.2	36.1 to 69.6	−0.45	−0.82 to − 0.09

Phase 1 was before the initiation of cytoreductive surgery (CRS) in the operation room, phase was 2 during CRS, phase 3 was during hyperthermic intraperitoneal chemotherapy (HIPEC) and phase 4 was after HIPEC before leaving to the intensive care unit

*LOA* Limits of agreement

**Fig. 2:**
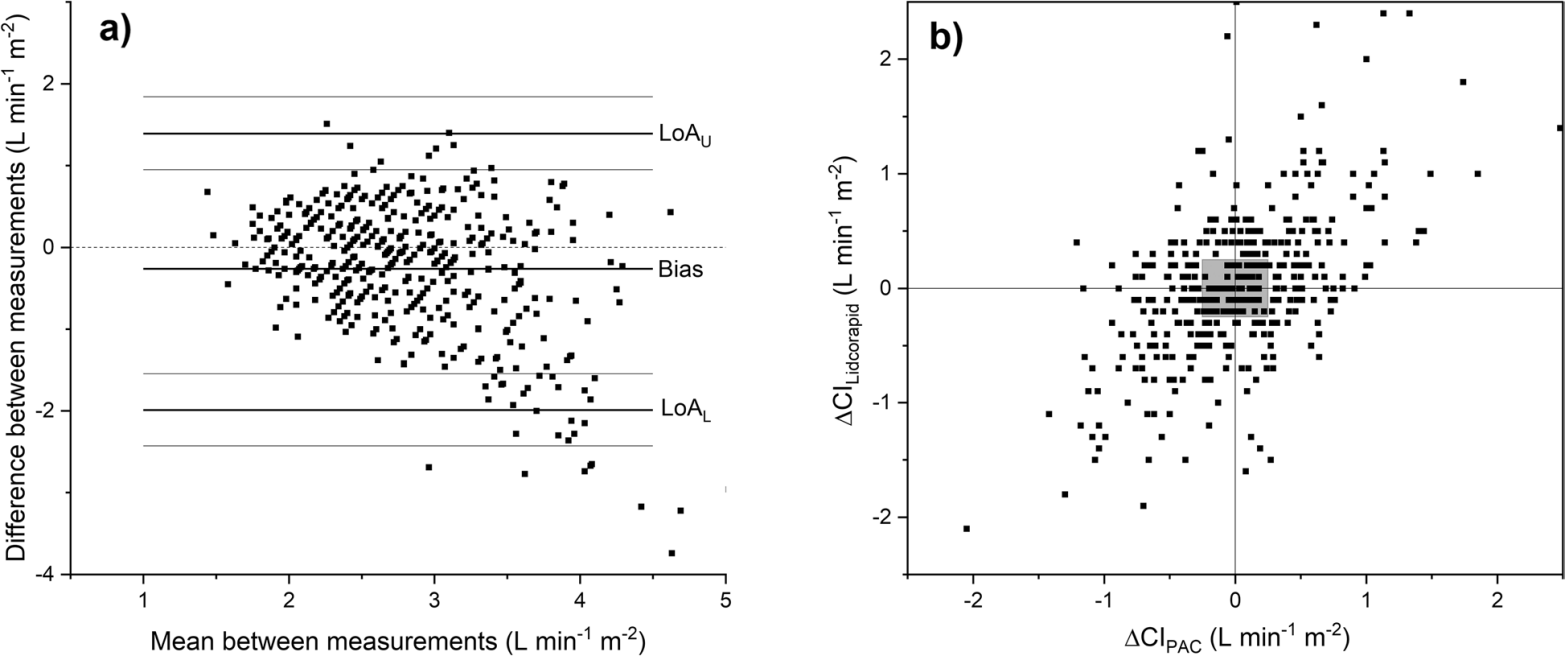
**a** The Bland-Altman analysis for cardiac index measurements with pulse power device LiDCOrapid and the bolus thermodilution technique with a pulmonary catheter, all measurement points. The bias and LOA with 95% CIs are shown with lines. Exact numbers are seen in Table 3. **b** The trending ability of LiDCOrapid evaluated with four-quadrant plot at all measurement points. The changes in consecutive cardiac index measured with LiDCOrapid (ΔCI_Lidcorapid_) and the bolus thermodilution technique (ΔCI_PAC_) are plotted against each other

LiDCOrapid compared with TDCO was associated with a bias of − 0.26 l min^− 1^ m^− 2^ (95% CI − 0.34 to − 0.18) at all measurement points. Standard deviation of the differences was 0.81. LOA were − 1.99 to 1.39 l min^− 1^ m^− 2^ and PE was 57.1% when all measurement points were regarded (Fig. 2a). The regression coefficient was − 0.36 l min^− 1^ m^− 2^. However, at phase 3 the bias was − 0.41 l min^− 1^ m^− 2^. Regression coefficient was statistically significant in the subgroup analysis at each phase, and at phases 1 and 3 the regression coefficient was nonlinear. In the 4Q plot the changes in *CI* measured by LiDCOrapid and TDCO were plotted against each other (Fig. 2b). The level of agreement in trending was assessed with error grids resulting in 35.2% of the measurement points being in zone 1. All the results between LiDCOrapid and TDCO are shown in Tables 3 and 4.

**Table 3:** Results from cardiac index measurements made by LiDCOrapid compared to bolus thermodilution technique with a pulmonary artery catheter

LiDCO rapid	Bias (l min^− 1^ m^− 2^)	Bias 95% CI	LOA lower (l min^− 1^ m^− 2^)	LOA lower 95% CI	LOA upper (l min^− 1^ m^− 2^)	LOA upper 95% CI	Percentage error (%)	Percentage error 95% CI	Regression coefficient (l min^− 1^ m^− 2^) ^a^ (l min^− 1^ m^− 2^) ^2 b^	Regression coefficient 95% CI
All *n* = 430	−0.26	−0.34 to − 0.18	− 1.99	−2.43 to − 1.55	1.39	0.95 to 1.84	57.1	42.8 to 74.2	−0.36 ^a^	− 0.44 to − 0.27
Phase 1 *n* = 29	−0.13	− 0.31 to 0.06	− 1.25	− 1.66 to − 0.84	0.99	0.57 to 1.40	46.1	31.1 to 68.1	−5.72 ^a^ 1.20 ^**b**^	− 10.34 to − 1.10 0.17 to 2.22
Phase 2 *n* = 268	−0.24	− 0.33 to − 0.14	−1.82	−2.26 to − 1.38	1.40	0.96 to 1.84	56.5	43.1 to 74.7	−0.34 ^a^	− 0.45 to − 0.24
Phase 3 *n* = 64	−0.41	− 0.66 to − 0.17	− 2.76	−3.52 to − 2.00	1.71	0.95 to 2.47	60.1	41.9 to 88.6	3.78 ^a^ − 0.51 ^**b**^	1.48 to 6.08 − 0.84 to − 0.18
Phase 4 *n* = 69	−0.24	− 0.43 to − 0.05	− 2.32	−2.95 to − 1.69	1.48	0.85 to 2.11	54.5	36.9 to 79.6	−0.65 ^a^	− 0.87 to − 0.43

Phase 1 was before the initiation of cytoreductive surgery (CRS) in the operation room, phase was 2 during CRS, phase 3 was during hyperthermic intraperitoneal chemotherapy (HIPEC) and phase 4 was after HIPEC before leaving to the intensive care unit

*LOA* Limits of agreement

^a^linear regression

^b^ quadratic term

**Table 4:** The error grid with four zones demonstrating the level of agreement in cardiac index when comparing both Starling SV and LiDCOrapid with bolus thermodilution technique with a pulmonary artery catheter. The trending ability is optimal in zone 1, whereas in zone 4 the trending ability is poor

	Starling SV					LiDCO rapid				
Error grid zone	All *n* = 416	Phase 1 *n* = 12	Phase 2 *n* = 270	Phase 3 *n* = 64	Phase 4 *n* = 70	All *n* = 402	Phase 1 *n* = 8	Phase 2 *n* = 262	Phase 3 *n* = 63	Phase 4 *n* = 69
Zone 1	32.5%	16.7%	40.7%	18.8%	15.7%	35.2%	37.5%	40.5%	20.6%	27.5%
Zone 2	14.4%	33.3%	13.7%	9.4%	18.6%	14.9%	12.5%	13.7%	17.5%	17.4%
Zone 3	34.4%	16.7%	31.5%	40.6%	42.9%	34.3%	37.5%	28.6%	49.2%	42.0%
Zone 4	18.8%	33.3%	14.1%	31.3%	22.9%	15.7%	12.5%	17.2%	12.7%	13.0%

Phase 1 was before the initiation of cytoreductive surgery (CRS) in the operation room, phase was 2 during CRS, phase 3 was during hyperthermic intraperitoneal chemotherapy (HIPEC) and phase 4 was after HIPEC before leaving to the intensive care unit

## Discussion

This study was performed to assess the reliability of the less invasive *CO* monitors, Starling SV and LiDCOrapid, compared with TDCO during CRS with HIPEC. The bias of both monitors over all phases was acceptable showing satisfactory accuracy. However, in a subgroup analysis on separate phases the bias increased and significant regression coefficients were present indicating that the bias was proportional to changes in *CI*. Neither of the monitors was precise, since the LOA were wide and the PE was high. The trending ability of both devices was insufficient, as less than 50% of the changes in *CI* were comparable to those measured with TDCO. These results limit the reliability of the devices in the setting of CRS with HIPEC.

There are a lot of hemodynamic changes during HIPEC due to increased temperature, such as increased heart rate, increased *CI*, increased oxygen consumption and decreased systemic vascular resistance [24]. Bleeding results in hypovolemia, as well as the major surgical resections, physicochemical trauma and HIPEC by altering the capillary permeability [2, 24]. While optimal fluid therapy often necessitates aggressive fluid resuscitation, avoiding fluid overdose is important [2]. In literature, goal-directed therapy (GDT) in CRS with HIPEC as well as in other major surgeries is associated with a better outcome, and monitoring *CI* is recommended to be routinely used during CRS with HIPEC to guide GDT and fluid administration [1–3, 25–27].

An ideal *CO* monitor would be reliable, noninvasive, continuous, cost-effective, operator independent and have a fast response time [28]. It is important to investigate the reliability of new *CO* monitors by comparing them with an accurate and precise reference method [29]. According to our knowledge, there are no previous studies comparing the bioreactance method or pulse power analysis to TDCO during CRS with HIPEC, since most studies on these devices assess cardiac patients.

An earlier version of bioreactance monitor (NICOM®) was compared with another accepted reference method transpulmonary thermodilution (TPTD) during CRS without HIPEC in patients with ovarian carcinoma [7, 30]. The accuracy and trending ability were acceptable, but precision was poor. The sample size was small, so the results cannot be directly compared with ours. Two other studies on NICOM showed insufficient reliability of the monitor, but the patients were nonsurgical [12, 31].

LiDCOrapid was compared with TDCO in patients after liver transplantation [32]. The results were similar to ours with acceptable accuracy, but imprecision and poor trending ability. In a study including patients undergoing either cardiac or hepatic surgery, LiDCOrapid showed inaccuracy and imprecision compared to TDCO. However, the sample size was small compared with ours (149 measurements) [33].

Previous studies comparing Starling SV or LiDCOrapid to TDCO or TPTD in cardiac patients have not shown sufficient reliability [34–37]. One study investigated postoperative cardiac surgery patients with impaired left ventricular function, and the *CO* was measured both with LiDCOplus and TDCO [38]. The results showed good accuracy and better precision than ours, but as LiDCOplus is calibrated with lithium, the results cannot be compared with ours.

A strength of our study is that we used highly recommended statistical methods. The Bland-Altman plot was used to assess the reliability of the monitors [7]. Since there are no specific reference values for acceptable bias or LOA, we set the targets according to the literature and according to our best clinical evaluation [7]. Concerning the PE, we used the acceptable value of 30% according to Critchley and Critchley since we had the recommended TDCO as our reference method [22]. As for trending ability, there are no preset ideal target values to interpret the results of 4Q plot or error grid. Hence, we did not have reference values in advance, which can be seen as a weakness. With interpretation of our results we used our best clinical knowledge.

Ideally the precision of a refence monitor would be calculated within the study, which we failed to perform. However, the precision of TDCO is proved to be 20% when using three consecutive reliable measurements to calculate the average value [7, 13, 22, 29, 39]. We did not calibrate LiDCOrapid since we wanted to investigate its qualities as a noncalibrated device. During HIPEC, the baseline of the TDCO was uneven. The reason for that could be the temperature changes due to simultaneous heating with Belmont Hyperthermia Pump and cooling with external devices, or a mechanical interference from the Belmont Hyperthermia Pump. The problem was detected only in some patients during the HIPEC phase, so the potential effects on the reliability of TDCO concern only the phase 3 in our study. Additionally, as a special characteristic of HIPEC, the rise in patient blood temperature during HIPEC can overestimate the *CI* measured with TDCO, which needs to be considered [40].

One of the possible benefits of the new monitors is that they are continuous, which could offer a positive insight into trending due to a fast response time. Our study design may be considered suboptimal since our reference method is intermittent. The problem is, however, that none of the continuous monitors have been proven to be reliable enough to be used as a reference method [7]. This is a limitation that needs to be considered when interpretating our results.

In conclusion, bioreactance-based Starling SV and pulse power analyser LiDCOrapid were not interchangeable with TDCO. Based on these results, we cannot recommend the use of Starling SV and LiDCOrapid in monitoring hemodynamics and guiding fluid therapy during CRS with HIPEC.

## Data Availability

The datasets used and analyzed during the current study are available from the corresponding author on request.
